# Impact of Antihypertensive Treatment on Maternal and Perinatal Outcomes in Pregnancy Complicated by Chronic Hypertension: A Systematic Review and Meta‐Analysis

**DOI:** 10.1161/JAHA.117.005526

**Published:** 2017-05-17

**Authors:** Louise M. Webster, Frances Conti‐Ramsden, Paul T. Seed, Andrew J. Webb, Catherine Nelson‐Piercy, Lucy C. Chappell

**Affiliations:** ^1^ Women's Health Academic Centre King's College London St Thomas’ Hospital London United Kingdom; ^2^ Cardiovascular Division Department of Clinical Pharmacology King's College London British Heart Foundation Centre St Thomas’ Hospital London United Kingdom

**Keywords:** antihypertensive agent, hypertension, meta‐analysis, pregnancy, systematic review, Pregnancy, Hypertension, Treatment, Meta Analysis

## Abstract

**Background:**

Chronic hypertension complicates around 3% of all pregnancies. There is evidence that treating severe hypertension reduces maternal morbidity. This study aimed to systematically review randomized controlled trials of antihypertensive agents treating chronic hypertension in pregnancy to determine the effect of this intervention.

**Methods and Results:**

Medline (via OVID), Embase (via OVID) and the Cochrane Trials Register were searched from their earliest entries until November 30, 2016. All randomized controlled trials evaluating antihypertensive treatments for chronic hypertension in pregnancy were included. Data were extracted and analyzed in Stata (version 14.1). Fifteen randomized controlled trials (1166 women) were identified for meta‐analysis. A clinically important reduction in the incidence of severe hypertension was seen with antihypertensive treatment versus no antihypertensive treatment/placebo (5 studies, 446 women; risk ratio 0.33, 95%CI 0.19‐0.56; I^2^ 0.0%). There was no difference in the incidence of superimposed pre‐eclampsia (7 studies, 727 women; risk ratio 0.74, 95%CI 0.49‐1.11; I^2^ 28.1%), stillbirth/neonatal death (4 studies, 667 women; risk ratio 0.37, 95%CI 0.11‐1.26; I^2^ 0.0%), birth weight (7 studies, 802 women; weighted mean difference −60 g, 95%CI −200 to 80 g; I^2^ 0.0%), or small for gestational age (4 studies, 369 women; risk ratio 1.01, 95%CI 0.53‐1.94; I^2^ 0.0%) with antihypertensive treatment versus no treatment/placebo.

**Conclusions:**

Antihypertensive treatment reduces the risk of severe hypertension in pregnant women with chronic hypertension. A considerable paucity of data exists to guide choice of antihypertensive agent. Adequately powered head‐to‐head randomized controlled trials of commonly used antihypertensive agents are required to inform prescribing.

## Introduction

Chronic hypertension complicates around 3% of all pregnancies.^1^, ^2^ There is growing evidence that the incidence is rising with increasing maternal age and obesity.^2^, ^3^, ^4^, ^5^ The increased risks of adverse perinatal outcomes for pregnant women with chronic hypertension are well established.^6^, ^7^ In addition, controlling severe systolic hypertension has been recommended repeatedly by national and international guidance to reduce the risks of maternal morbidity and mortality.^8^, ^9^, ^10^

There remains some debate regarding the efficacy of treating chronic hypertension in pregnancy before it reaches severe levels due to concerns for fetal growth.^11^, ^12^, ^13^, ^14^, ^15^, ^16^ Internationally, guidelines vary for the management of chronic hypertension in pregnancy.^17^ However, the Control of Hypertension in Pregnancy Study, published in 2015, reported that there was no effect of tight blood pressure control (target diastolic 85 mm Hg) compared to less tight control (target diastolic 105 mm Hg) on a composite outcome of pregnancy loss and high‐level neonatal care within the first 48 hours of infant life (31.4% versus 30.7%) and the overall risk of small‐for‐gestational‐age infants (birth weight <10th centile) was not different between groups (16.1% versus 19.7%; odds ratio 0.78, 95%CI 0.56‐1.08). The frequency of severe hypertension was significantly higher with less‐tight control compared with tight control (40.6% versus 27.5%; odds ratio 1.8, 95%CI 1.3‐2.4).^18^ There are likely to be additional benefits of reducing the incidence of severe hypertension through a decrease in short‐ and long‐term maternal morbidity and mortality from stroke and other end‐organ damage^9^, ^19^, ^20^, ^21^, ^22^ and potential cost savings with a reduction in healthcare resource use.^23^, ^24^

Given the physiological demands of pregnancy, duration of treatment and potential impacts on maternal and perinatal outcomes, there is a need for evidence on efficacy and safety of antihypertensive treatment specifically in pregnancy complicated by chronic hypertension. Current international guidance points to the lack of evidence for antihypertensive agent prescribing in chronic hypertension in pregnancy.^8^, ^17^ Because the benefits of tight‐control blood pressure targets have now been demonstrated in women with hypertension in pregnancy, this study aimed to systematically review and meta‐analyze available data from randomized controlled trials specifically in chronic hypertension to establish the efficacy and safety of antihypertensive agents or class of agents.

## Methods

The study protocol for this systematic review was developed in line with the PRISMA‐P statement^25^ and registered on the PROSPERO database (http://www.crd.york.ac.uk/PROSPERO/ reference number CRD42015020733). No ethical approval was required.

### Literature Search

A comprehensive literature review using Medline (via Ovid), Embase (via Ovid), and the Cochrane Trials Register from their earliest entries until the November 30, 2016 was performed. Search strategies were adapted to each database. Searches of exploded MeSH terms “pregnancy,” “hypertension,” and “antihypertensive” (Embase) or “cardiovascular agent” (Medline) were performed individually and then combined in each database. For Medline and Embase searches, a search filter for randomized controlled trials was then applied as recommended in the *Cochrane Handbook for Systematic Reviews of Interventions*.^26^ Relevant unpublished data were sought by searching for trials registered on clinicaltrials.gov and ISRCTN (www.isrctn.com) and reviewing thesis titles from the World Cat dissertations and theses database. References of retrieved studies and relevant review articles were also searched using the snowballing approach. No language restrictions were applied. The study protocol (including the literature search strategy) is detailed in Data S1.

### Study Selection Criteria

All randomized controlled trials of pregnant women with chronic hypertension comparing an antihypertensive agent with another treatment arm as long‐term antepartum management were included. No blood pressure cutoffs were utilized in the eligibility criteria for inclusion, but studies examining acute treatment of severe hypertension via intravenous/fast‐acting routes were excluded. Comparisons with other antihypertensive drug(s), placebo, no treatment, or an alternative such as bed rest were eligible for inclusion. Studies that included participants with gestational hypertension and chronic hypertension were only eligible for inclusion if the data for the women with chronic hypertension were reported separately to allow fair comparison. Studies that compared management strategies only but did not include a randomized comparison of drug treatments were not eligible for inclusion. Trials that did not report any of the predefined outcomes were excluded. Trials that did not include sufficient information on the outcomes (eg, standard deviations) could not be included in the meta‐analysis. No other restrictions were applied to the study search.

### Data Extraction

The titles, abstracts and selected full texts generated from the literature search were independently screened by authors L.M.W. and F.C.R. Data from the trials that met all inclusion criteria were manually extracted and entered into a standard extraction table independently from full texts by L.M.W. and F.C.R. The authors were not masked to the results of the study or authors. Where 2 articles published results from the same study, individual pertinent outcomes were extracted from both articles without repetition of data extraction. The following outcome measures were recorded for each study: maternal, severe hypertension (definitions used in each study documented), superimposed pre‐eclampsia (definitions used in each study documented), cesarean section delivery, abruption; perinatal, stillbirth/neonatal death, birth weight, small‐for‐gestational‐age infants (within trial definition), preterm birth (defined as less than 37 completed weeks’ gestation), and Apgar score less than 7 at 5 minutes. Details of potential confounders (maternal age, body mass index, ethnicity) were recorded wherever provided in the manuscripts. The PRISMA statement was considered and observed for all procedures and reporting.^27^

### Study Quality Assessment

Each trial was independently quality assessed using the Cochrane Collaboration Risk of Bias tool by L.M.W. and F.C.R.^26^ The risk of bias in each of the following domains was assessed: sequence generation, allocation concealment, blinding, incomplete outcome data, selective outcome reporting, and other sources of bias.

### Statistical Methods

Data were analyzed in the statistical package Stata (version 14.1, StataCorp, College Station, TX), using the metan suite of commands.^28^ All outcomes were analyzed on an intention‐to‐treat basis. Per‐protocol data for an end point were excluded from the analysis. Meta‐analysis was performed using a fixed‐effects model where there was more than 1 study with analyzable data. If there was evidence of significant heterogeneity, the meta‐analysis was repeated using the random‐effects model for comparison; however, the results presented are from the fixed‐effects analysis. Initial analysis of treatment effects was performed by class of antihypertensive agent and subsequently by active versus nonactive treatment. Treatment effects are presented as estimated differences in mean or risk ratios with 95% confidence intervals. Heterogeneity was quantified via the τ‐squared and I‐squared statistics.^29^

## Results

### Description of Studies

**Figure 1:**
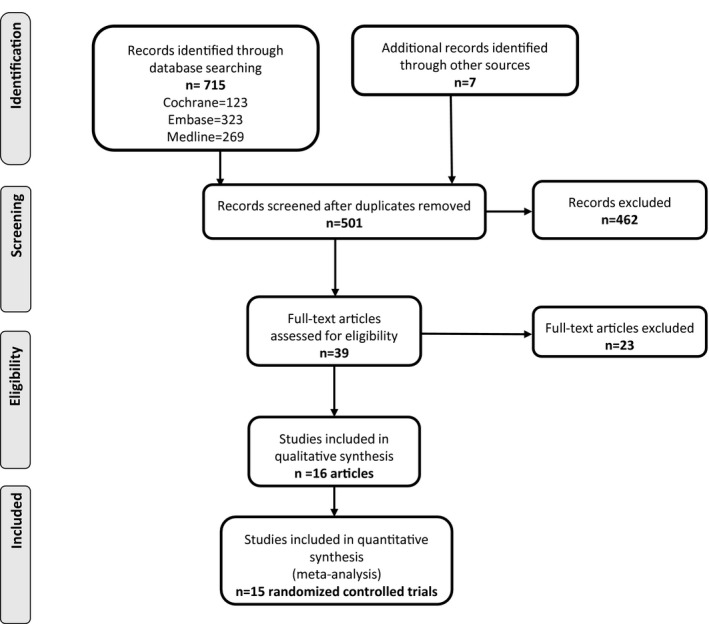
Flowchart of articles identified reporting randomized controlled trials of antihypertensive agents for the treatment of chronic hypertension in pregnancy.

The study selection process is illustrated in the flowchart (Figure 1). After removal of duplicates, the initial search generated 501 titles and abstracts for review. Following screening, 39 articles underwent full‐text assessment. Sixteen articles met inclusion criteria, reporting on 15 trials that recruited a total of 1166 women, with a median of 20 participants per trial (interquartile range 12‐60 participants per trial). The characteristics of the studies meeting entry criteria are presented in Table 1.

**Table 1:** Characteristics of the Studies Included in the Meta‐Analysis

Study First Author, Country, Year	Methods	Participants With Chronic Hypertension	Intervention	Outcomes Included in Meta‐Analysis
Arias, USA, 1979^30^, ^a^	Participants allocated randomly to antihypertensive treatment or no treatment No allocation concealment	58 women History of hypertension before pregnancy (BP >140/90 mm Hg) OR hypertension in 2 consecutive measurements more than 24 h apart at <20 weeks’ gestation AND diastolic BP <100 mm Hg and no end organ damage *Excluded:* Nulliparous women Major obstetric or medical problem, eg, diabetes mellitus >20 weeks’ gestation	*Active:* A varied combination of: Methyldopa 750 to 2000 mg/day AND/OR hydralazine 75 to 250 mg/day AND/OR hydrochlorothiazide 50 mg/day *Vs nonactive:* No treatment	*Maternal:* Severe hypertension Superimposed pre‐eclampsia Mode of delivery *Perinatal:* Stillbirth/neonatal death Birth weight Preterm birth
Butters, UK, 1990^31^	Double‐blind randomized controlled trial	29 women Systolic BP 140 to 170 mm Hg OR diastolic BP 90 to 110 mm Hg on 2 occasions separated by at least 24 h between 12 and 24 weeks’ gestation in women with known essential hypertension *Excluded:* Contra‐indication to β‐blocker	*Active:* Atenolol 50 to 200 mg/day *Vs nonactive:* Placebo tablets	*Perinatal:* Stillbirth Birth weight Small for gestational age
Fiddler, UK, 1983^32^	Participants mixed population of gestational and chronic hypertension Stratified randomization Open label	46 women Diastolic BP >95 mm Hg on 2 occasions at least 24 h apart <32 weeks’ gestation OR diastolic BP >105 mm Hg on 1 occasion at <32 weeks’ gestation *Excluded:* Diabetes mellitus Multiple pregnancy Already taking antihypertensive treatment Significant medical condition	*Active:* Methyldopa 750 to 3000 mg/day *Vs active:* Oxprenolol 160 to 640 mg/day	*Maternal:* Severe hypertension Mode of delivery *Perinatal:* Birth weight Apgar score <7 at 5 min
Freire, Brazil, 1988^33^	Consecutive randomization allocation No information on allocation concealment	40 women Known chronic hypertension Diastolic BP >95 mm Hg *Excluded:* Proteinuria at study entry End‐organ disease	*Active:* Methyldopa 250 to 2000 mg/day *Vs active:* Pindolol 10 to 30 mg/day	*Maternal:* Severe hypertension Superimposed pre‐eclampsia *Perinatal:* Stillbirth Birth weight Apgar score <7 at 5 min
Hirsch, Israel, 1996^34^	Randomized using serial numbers in blocks of 6 No information on allocation concealment	27 women Elevated BP before pregnancy or diastolic BP 85 to 99 mm Hg at <20 weeks’ gestation *Excluded:* Known medical or obstetric complication that could affect pregnancy outcome β‐blockers contraindicated	*Active:* Pindolol 10 to 20 mg/day *Vs non‐active:* Placebo tablets	*Maternal:* Severe hypertension *Perinatal:* Birth weight Small for gestational age Apgar score <7 at 5 min
Horvath, Australia, 1985^35^	Participants mixed population of gestational and chronic hypertension Double‐blind, randomized trial Participants entered in numerical sequence	16 women Known essential hypertension OR failure of hypertension to resolve 12 weeks postpartum from previous pregnancy OR BP >130/85 mm Hg on 2 or more occasions	*Active:* Methyldopa 250 to 2000 mg/day *Vs nonactive:* Clonidine 150 to 1200 μg/day	*Perinatal:* Stillbirth/neonatal death
Kahhale, Brazil, 1985^36^	Women divided into 2 groups—treatment and control No information regarding concealment	100 women BP >140/90 mm Hg before 20 weeks’ gestation *Excluded:* Proteinuria at study entry Contraindication to β‐blockers	*Active:* Pindolol 10 to 30 mg/day *Vs nonactive:* No treatment	*Perinatal:* Stillbirth Birth weight Apgar score <7 at 5 min
Mutch, UK, 1977^37^, ^b^	Participants mixed population of gestational and chronic hypertension Randomly allocated Open label	202 women BP >140/90 mm Hg on 2 occasions at least 24 h apart before 28 weeks’ gestation *Excluded:* BP at study entry >170 mm Hg systolic or >110 mm Hg diastolic Multiple pregnancy Rhesus incompatibility Severe maternal disease	*Active:* Methyldopa—dosing regimen not specified *Vs nonactive:* No treatment	*Maternal:* Severe hypertension Superimposed pre‐eclampsia Mode of delivery
Parazzini, Italy, 1998^38^	Participants mixed population of gestational and chronic hypertension Computer‐generated randomization list Open label	126 women Known chronic hypertension before pregnancy with 2 consecutive diastolic BP >90 mm Hg OR diastolic BP >90 mm Hg before 20 weeks’ gestation *Excluded:* Chronic disease, eg, diabetes mellitus, renal disease Fetal malformations Already on antihypertensive treatment Contraindications to nifedipine	*Active:* Nifedipine slow release 20 to 80 mg/day *Vs nonactive:* No treatment	*Perinatal:* Birth weight
Redman, UK, 1976^39^, ^b^	Participants mixed population of gestational and chronic hypertension Allocated randomly to treatment group Open label	208 women BP >140/90 mm Hg on 2 occasions at least 24 h apart before 28 weeks’ gestation *Excluded:* Severe hypertension at study entry (systolic BP >170 mm Hg or diastolic BP >110 mm Hg) Already on antihypertensive treatment Multiple pregnancy Diabetes mellitus Rhesus immunisation	*Active:* Methyldopa—dosing regimen not specified *Vs nonactive:* No treatment	*Perinatal:* Stillbirth/neonatal death Birth weight
Sibai, USA, 1984^40^	Participants taking diuretics randomized to continue or discontinue treatment Open label	20 women Long‐term history of hypertension, diastolic BP >90 and <110 mm Hg Receiving diuretics before pregnancy	*Active:* Diuretics—specific agent(s) and doses not specified *Vs nonactive:* No treatment (diuretics discontinued)	*Maternal:* Superimposed pre‐eclampsia Mode of delivery *Perinatal:* Birth weight Preterm birth Apgar score <7 at 5 min
Sibai, USA, 1990^41^	Computer‐generated randomization via list of numbers Open label	263 women History of chronic hypertension prior to pregnancy *Excluded:* Medical complications other than chronic hypertension	*Active:* Methyldopa 750 to 4000 mg/day *Vs active:* Labetalol 300 to 2400 mg/day *Vs nonactive:* No treatment	*Maternal:* Superimposed pre‐eclampsia Mode of delivery Abruption *Perinatal:* Stillbirth/neonatal death Birth weight Preterm birth Apgar score <7 at 5 min
Steyn, South Africa, 1997^42^	Double‐blind randomized placebo‐controlled trial Computer‐generated randomization numbers, using balanced‐block method	138 women Diastolic BP persistently >80 mm Hg between 12 and 20 weeks’ gestation without proteinuria *Excluded:* Multiple pregnancy Bradycardia on ECG	*Active:* Ketanserin 40 to 80 mg/day *Vs nonactive:* Placebo tablets	*Maternal:* Severe hypertension Superimposed pre‐eclampsia Abruption *Perinatal:* Stillbirth/neonatal death
Voto, Argentina, 1990^43^	Participants mixed population of gestational and chronic hypertension Randomized comparative study Open label	49 women Known chronic hypertension with BP >159/99 mm Hg twice 24 h apart *Excluded:* Women requiring more than 1 drug to control BP	*Active:* Atenolol 50 to 200 mg/day *Vs active:* Methyldopa 500 to 2000 mg/day *Vs active:* Ketanserin 80 to 120 mg/day	*Maternal:* Superimposed pre‐eclampsia
Weitz, USA, 1987^44^	Double blind randomized study	25 women Chronic hypertension, BP 140/90 mm Hg on 2 occasions >6 h apart No proteinuria Singleton pregnancies <34 weeks’ gestation	*Active:* Methyldopa 750 to 2000 g/day *Vs nonactive:* Placebo tablets	*Maternal:* Superimposed pre‐eclampsia *Perinatal:* Stillbirth/neonatal death
Welt, USA, 1981^45^	Prospective cohort study with subgroup randomized to treatment Not clear if either clinician and/or participant blinded to treatment allocation	21 women With documented prepregnancy history of elevated BP >140/90 mm Hg on 2 occasions >6 h apart OR in first 2 trimesters of pregnancy OR undocumented history of hypertension for which the patient was taking antihypertensive treatment before or during pregnancy *Excluded:* Diabetes mellitus requiring insulin Multiple pregnancy Planning to terminate pregnancy	*Active:* Methyldopa 750 mg/day—maximum dose not given *Vs active:* Hydralazine 75 mg/day—maximum dose not given *Vs nonactive:* Placebo tablets	*Maternal:* Severe hypertension Superimposed pre‐eclampsia *Perinatal:* Small for gestational age

BP indicates blood pressure.

a Participants randomized to antihypertensive treatment (not to an agent) vs no antihypertensive treatment.^30^

b Articles reporting on the same study population.

All studies included in the meta‐analysis were completed before 1998. Ten of the trials were conducted in a predefined chronic hypertension cohort alone,^1^ and the remaining 5 reported outcomes for a subgroup of women with chronic hypertension.^32^, ^35^, ^37^, ^38^, ^39^, ^43^ Six studies were head‐to‐head comparisons of 2 or more antihypertensive agents (435 women),^32^, ^33^, ^35^, ^41^, ^43^, ^45^ 4 were placebo‐controlled studies of a single antihypertensive agent (219 women),^31^, ^34^, ^42^, ^44^ and 5 were studies of a single antihypertensive agent compared to no treatment (714 women).^30^, ^36^, ^37^, ^38^, ^39^, ^40^

Of the 23 articles that were excluded, 14 studies included a mixed population of gestational and chronic hypertension and did not report outcomes separately, 6 studies included only gestational hypertension, 1 article reported no additional outcomes for a trial already included in the meta‐analysis (Table 2).^46^, ^47^, ^48^, ^49^, ^50^, ^51^, ^52^, ^53^, ^54^, ^55^, ^56^, ^57^, ^58^, ^59^, ^60^, ^61^, ^62^, ^63^, ^64^, ^65^, ^66^, ^67^, ^68^ In addition, Leather and colleagues reported a randomized controlled trial in 1968 that recruited 47 chronic hypertensive participants randomized to bendroflumethiazide and methyldopa versus no treatment. This article could not be included due to inadequate reporting of the statistical information relating to the outcomes, prohibiting inclusion of the data in the meta‐analysis.^58^ Leather and colleagues concluded that the treatment of “early hypertension” (present before 20 weeks’ gestation) resulted in a longer pregnancy, increased birth weight and reduced perinatal mortality. A pilot study by Vigil‐De Gracia and colleagues in 2014 compared furosemide, amlodipine, and aspirin in a 3‐arm randomized controlled trial and found no significant difference in outcomes among all treatment arms.^65^ These data could not be included in the active versus nonactive treatment meta‐analysis, as the third arm of aspirin was considered active treatment given that the other arms did not receive this agent. In addition, the data from the amlodipine and furosemide arms could not be included in the antihypertensive treatment versus antihypertensive treatment meta‐analysis as there are no other head‐to‐head trials evaluating calcium‐channel blockers or diuretics for comparison.

**Table 2:** Studies Excluded From the Meta‐Analysis and Rationale

Study (First Author, Country, Year Published)	Reason for Exclusion and Study Details
Antony, South Africa, 1990^46^	Study participants had gestational and not chronic hypertension *Methods:* Prospective, randomized block design, no further details given *Participants:* 60 women at 28 to 36 weeks’ gestation with mean 24‐h diastolic BP 100 to 120 mm Hg±proteinuria *Intervention:* Indoramin 50 mg twice daily vs methyldopa 1 g twice daily vs placebo 1 tablet daily
Bolte, Netherlands, 1998^47^	Study participants had gestational or chronic hypertension. Outcomes for those with chronic hypertension not reported separately *Methods:* Randomized, open‐label multicenter trial *Participants:* 31 women, 26 to 32 weeks’ gestation with diastolic BP >110 mm Hg and previously normotensive or in women with chronic hypertension: diastolic BP >20 mm Hg compared to BP at <20 weeks. *Intervention:* IV ketanserin 5 mg bolus then 4 mg/h vs IV dihydralazine 1 mg/h
Bott‐Kanner, Israel, 1992^48^	Study participants had gestational or chronic hypertension. Outcomes for those with chronic hypertension not reported separately *Methods:* Randomized, double‐blind trial. Women randomized in blocks of 6 using serial numbers *Participants:* 60 women before 35 weeks’ gestation with diastolic BP 85 to 99 mm Hg *Intervention:* Pindolol 5 mg twice daily or placebo 1 tablet twice daily
Cruickshank, UK, 1991^49^	Study participants had gestational and not chronic hypertension *Methods:* Randomized open‐label trial, using numbered sealed envelopes *Participants:* 114 women with singleton pregnancies between 24 and 39 weeks’ gestation, diastolic BP >90 mm Hg for >24 h in absence of proteinuria *Intervention:* Labetalol 100 mg twice daily vs no treatment
Faneite, Venezuela, 1988^50^	Study participants had gestational or chronic hypertension. Outcomes for those with chronic hypertension not reported separately *Methods:* Randomized trial *Participants:* 31 women >14 weeks’ gestation, with BP >140/90 and <170/110 mm Hg on 2 occasions *Intervention:* Mepindolol 5 mg once daily vs methyldopa 250 mg twice daily
Gallery, Australia, 1979^51^	Study participants had gestational or chronic hypertension. Outcomes for those with chronic hypertension not reported separately *Methods:* Randomized comparison study *Participants:* 56 women at any gestation with sitting diastolic BP >95 mm Hg on 2 occasions at least 24 h apart or 100 mm Hg on 2 occasions at least 8 h apart *Intervention:* Oxprenolol vs methyldopa. Doses not specified
Gallery, Australia, 1985^52^	Study participants had gestational or chronic hypertension. Outcomes for those with chronic hypertension not reported separately *Methods:* Randomized open study, allocation by random number series *Participants:* 183 women with singleton pregnancies and sitting diastolic BP of >90 mm Hg on 2 occasions at least 24 h apart or >95 mm Hg on 2 occasions 12 h apart or >100 mm Hg on 2 occasions 8 h apart *Intervention:* Oxprenolol 40 mg twice daily vs methyldopa 250 mg twice daily
Hall, South Africa, 2000^53^	Study participants had pre‐eclampsia or gestational or chronic hypertension. Outcomes for those with chronic hypertension not reported separately *Methods:* Randomized single‐blind controlled trial. Computer‐generated balanced blocks of 50 numbers. Women allocated using consecutive numbered, opaque envelopes containing medication *Participants:* 150 women with severe early‐onset pre‐eclampsia or hypertension and BP not controlled with methyldopa 2 mg daily *Intervention:* Nifedipine 10 mg 3 times daily vs prazosin 1 mg 3 times daily
Henderson‐Smart, Australia, 1984^54^	Details of participants with chronic hypertension not stated *Methods:* Reporting neonatal outcomes of infants born to women with hypertension in pregnancy who were entered in a prospective randomized double‐blind trial *Participants:* 95 infants born to mothers treated with clonidine hydrochloride and methyldopa *Intervention:* Clonidine hydrochloride 150 to 1200 μg/day vs methyldopa 250 to 2000 mg/day
Högstedt, Sweden, 1985^55^	Study participants had gestational or chronic hypertension. Outcomes for those with chronic hypertension not reported separately *Methods:* Randomized open controlled trial *Participants:* 161 women in antenatal care with diastolic BP >90 mm Hg on 2 occasions at least 6 h apart, confirmed the following day with diastolic BP >90 mm Hg for at least 2 out of 4 BP readings *Intervention:* 50 mg metoprolol and 25 mg hydralazine twice daily vs no treatment
Jannet, France, 1994^56^	Study participants had gestational or chronic hypertension or pre‐eclampsia. Outcomes for those with chronic hypertension not reported separately *Methods:* Randomized comparative trial. Computer‐generated random numbers, allocated using sealed envelopes *Participants:* 100 women with singleton pregnancies at >20 weeks’ gestation with systolic BP >140 mm Hg and/or diastolic BP >90 mm Hg on 2 successive measurements *Intervention:* Nicardipine 20 mg 3 times daily vs slow‐release metoprolol 200 mg once daily
Lardoux, France, 1988^57^	Study participants had gestational or chronic hypertension. Outcomes for those with chronic hypertension not reported separately *Methods:* Randomized open comparative trial *Participants:* 63 women between 7 and 36 weeks’ gestation with diastolic BP >90 mm Hg on 2 occasions at least 8 days apart *Intervention:* Methyldopa 500 mg/day vs labetalol 400 mg/day vs acebutolol 400 mg/day
Leather, UK, 1968^58^	Outcome data not presented with adequate statistical information to allow inclusion in the meta‐analysis *Methods:* Randomized controlled trial *Participants:* 100 women with diastolic BP >90 mm Hg on 2 occasions at least 48 h apart *Intervention:* Bendroflumethiazide 5 to 10 mg daily and methyldopa 400 to 2000 mg daily vs no treatment
Livingstone, Australia, 1983^59^	Study participants had gestational and not chronic hypertension *Methods:* Randomized prospective study, no further details given *Participants:* 28 women with BP >140/90 mm Hg on 2 consecutive readings at least 24 h apart *Intervention:* Propranolol vs methyldopa. Doses not specified
Moore, UK, 1982^60^	Study participants had gestational or chronic hypertension. Outcomes for women with chronic hypertension not reported separately *Methods:* Randomized trial, no further details given *Participants:* 74 women at <36 weeks’ gestation with systolic BP >170 mm Hg and/or diastolic BP >110 mm Hg *Intervention:* Labetalol 100 mg 4 times daily vs 250 mg methyldopa 4 times daily
Plouin, France, 1988^61^	Study participants had gestational or chronic hypertension. Outcomes for women with chronic hypertension not reported separately *Methods:* Randomized open controlled trial. Stratified randomization using blinded envelopes *Participants:* 176 women with a singleton pregnancy, gestational age between 12 and 34 weeks and diastolic BP >89 mm Hg on 2 separate occasions *Intervention:* Labetalol 400 mg in 2 doses vs methyldopa 500 mg in 2 doses
Rosenfeld, Israel, 1986^62^	Study participants had gestational or chronic hypertension. Outcomes for those with chronic hypertension not reported separately *Methods:* Randomized study, no further details given *Participants:* 44 women at <36 weeks’ gestation with systolic BP >150 mm Hg or diastolic BP >90 mm Hg on 2 separate occasions at least 24 h apart *Intervention:* Hydralazine 25 mg twice daily vs hydralazine 25 mg twice daily and pindolol 5 mg twice daily
Steyn, South Africa, 2001^63^	Reporting data from same trial as Steyn 1997,^42^ reported no additional outcomes *Methods:* Randomized double blind controlled trial. Computer‐generated balanced‐block structure *Participants:* 102 women between 12 and 20 weeks’ gestation with diastolic BP >80 mm Hg without proteinuria *Intervention:* Ketanserin 20 mg twice daily and aspirin 75 mg once daily vs placebo 1 tablet twice daily and aspirin 75 mg once daily
Tuimala, Finland, 1988^64^	Study participants had gestational and not chronic hypertension *Methods:* Randomized trial, no further details given *Participants:* 51 women with BP >149/94 mm Hg measured twice in sitting position after 2 days’ bed rest in hospital *Intervention:* Atenolol 50 to 100 mg/day vs pindolol 10 to 20 mg/day. If needed, hydralazine 150 mg/day added
Vigil‐De Gracia, Panama, 2014^65^	3‐arm pilot study including a third active treatment arm of aspirin *Methods:* Randomized open‐label pilot trial. Computer‐generated code with block size of 6, allocation through sealed envelopes *Participants:* 63 women at <20 weeks’ gestation with systolic BP 90 to 109 mm Hg *Intervention:* Furosemide 20 mg once daily vs amlodipine 5 mg once daily vs aspirin 75 mg once daily
Voto, Argentina, 1987^66^	Study participants had gestational or chronic hypertension or pre‐eclampsia. Outcomes for those with chronic hypertension not reported separately *Methods:* Randomized open study, no further details given *Participants:* 20 women with systolic BP >159 mm Hg and/or diastolic BP >99 mm Hg recorded twice 24 h apart *Intervention:* Ketanserin 20 to 80 mg daily vs methyldopa 500 to 2000 mg daily
Wichman, Sweden, 1984^67^	Study participants had gestational and not chronic hypertension *Methods:* Randomized placebo‐controlled trial. Selective allocation *Participants:* 52 women at <37 weeks’ gestation with systolic BP >140 mm Hg or diastolic BP >90 mm Hg or if there was an elevation of >30 mm Hg systolic or >15 mm Hg diastolic from previous readings *Intervention:* Metoprolol 50 mg twice daily vs placebo 1 tablet twice daily
Wide‐Swensson, Sweden, 1995^68^	Study participants had gestational and not chronic hypertension *Methods:* Randomized parallel double‐blind multicenter trial. Block randomization by numbers, allocation by sealed envelope *Participants:* 118 women with singleton pregnancy, gestational age between 25 and 37 weeks and diastolic BP between >95 and <110 mm Hg *Intervention:* Isradipine slow‐release 5 mg twice daily vs placebo 1 tablet twice daily

BP indicates blood pressure; IV, intravenous.

Definitions of severe hypertension and superimposed pre‐eclampsia for each included study are listed in Table 3. Minimum diastolic and systolic blood pressure eligibility cutoffs ranged from 80 to 99 and 140 to 160 mm Hg, respectively. Two studies excluded women with proteinuria,^33^, ^44^ 3 studies included women with proteinuria at study entry,^32^, ^35^, ^43^ and the remainder of studies did not specify presence or absence of proteinuria in their methods. Six studies excluded multifetal pregnancies^30^, ^32^, ^40^, ^41^, ^44^, ^45^; the remainder either included women with multifetal pregnancies or did not specify inclusion or exclusion in their methods. Maternal age was the only potential confounding baseline characteristic consistently reported. This ranged from 28 to 33 years, and no adjustment was deemed pertinent to this analysis. Body mass index was not reported in any of the trials, but 6 studies reported maternal weight at trial entry. Ethnicity of the participants was not considered or recorded in any of the trials.

**Table 3:** Definitions of Severe Hypertension and Superimposed Pre‐Eclampsia for Each Included Study

Study (First Author, Country, Year)	Definition of Severe Hypertension	Definition of Superimposed Pre‐Eclampsia
Arias, USA, 1979^30^	“Pregnancy‐aggravated hypertension”: >28 weeks’ gestation diastolic BP >100 mm Hg in 2 consecutive readings 6 or more h apart	>1+ proteinuria or more than 300 mg/L protein in 24‐h collection with “pregnancy‐aggravated hypertension” (see definition of severe hypertension)
Butters, UK, 1990^31^	Not reported	Not reported
Fiddler, UK, 1983^32^	Admitted to hospital for hypertension: diastolic BP >110 mm Hg	Not reported
Freire, Brazil, 1988^33^	Diastolic BP persistently >110 mm Hg	Systolic BP increased by 30 mm Hg or diastolic BP increased by 20 mm Hg for 2 consecutive readings at least 6 h apart OR proteinuria OR edema
Hirsch, Israel, 1996^34^	Uncontrolled elevation of diastolic BP >100 mm Hg	Not reported
Horvath, Australia, 1985^35^	Not reported	Not reported
Kahhale, Brazil, 1985^36^	Not reported	BP >170/110 mm Hg or proteinuria <37 weeks’ gestation
Mutch, UK, 1977^37^, ^a^	Systolic BP >170 mm Hg or diastolic BP >110 mm Hg on 2 occasions >4 h apart	Edema, proteinuria from midstream urine in absence of infection and raised plasma urate
Parazzini, Italy, 1998^38^	Not reported	Not reported
Redman, UK, 1976^39^, ^a^	Systolic BP >170 mm Hg or diastolic BP >110 mm Hg on 2 occasions >4 h apart	Edema, proteinuria from midstream urine in absence of infection and raised plasma urate
Sibai, USA, 1984^40^	Not reported	Not defined but reported as confirmed superimposed pre‐eclampsia
Sibai, USA, 1990^41^	Systolic BP >160 mm Hg or diastolic BP >100 mm Hg	Proteinuria (>1 g/24 h) or elevated uric acid (≥6 mg/dL) during second half of pregnancy
Steyn, South Africa, 1997^42^	Single diastolic BP >120 mm Hg OR 2 consecutive readings of 110 mm Hg at least 4 h apart	Single diastolic BP >110 mm Hg or 2 consecutive measurements of 90 mm Hg or more at least 4 h apart with proteinuria 300 mg/L on 24‐h collection OR 2+ proteinuria on dipstick
Voto, Argentina, 1990^43^	Not reported	Additional proteinuria
Weitz, USA, 1987^44^	Not reported	Sudden rise in systolic BP >30 mm Hg or diastolic BP >15 mm Hg and sudden weight gain >2 lb per week OR proteinuria 2+ or more on dipstick
Welt, USA, 1981^45^	Diastolic BP >100 torr on 2 occasions 6 or more h apart	Proteinuria >trace on dipstick or >300 mg/L in 24 h, edema, or both

BP indicates blood pressure.

a Articles reporting the same study population.

### Risk of Bias in Included Studies

**Figure 2:**
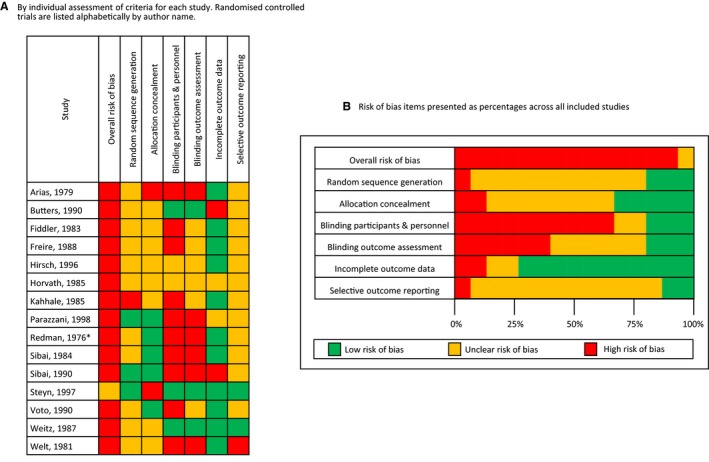
Risk‐of‐bias assessment of each study included in the meta‐analysis. A, Risk‐of‐bias assessment by individual assessment of criteria for each study. Randomized controlled trials are listed alphabetically by author name. B, Risk‐of‐bias items presented as percentages across all included studies. *Redman et al^39^ and Mutch et al^37^ both publish data from the same study; only the Redman article has been assessed for risk of bias. Risk‐of‐bias summary shows review authors’ judgments about each risk‐of‐bias domain in randomized controlled trials on efficacy of antihypertensive treatment for chronic hypertension in pregnancy.

All studies were assessed to be at high risk of bias apart from Steyn and colleagues,^42^ which was assigned unclear risk of bias. Full details of the allocated risk‐of‐bias scoring are displayed in Figure 2. No formal assessment of socioeconomic settings of the studies was made given the small number of studies, but all were from middle‐ or high‐income countries (see Table 1).

### Effects of Intervention: Active Versus Nonactive Treatment

**Figure 3:**
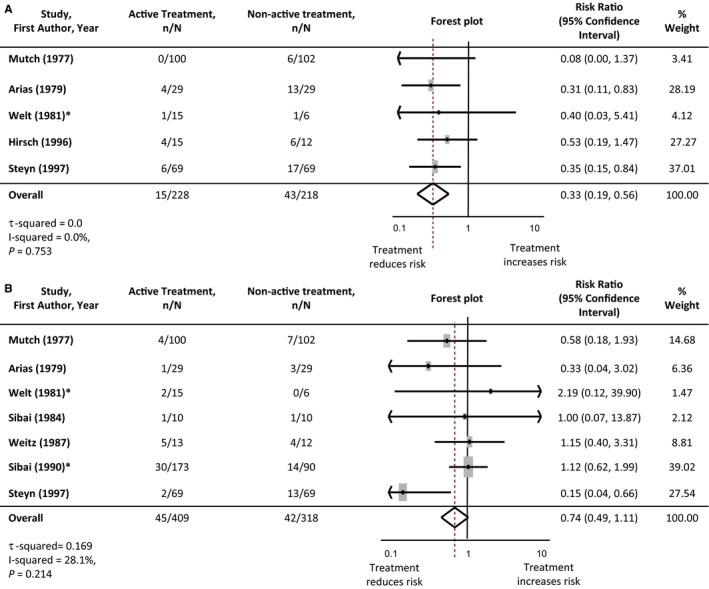
Maternal outcomes: active vs nonactive treatment. A, Severe hypertension. B, Superimposed pre‐eclampsia. *Where studies had more than 1 active treatment arm, the data from the active treatment arms were pooled and compared with the non‐active‐treatment data. Studies are listed in order of the year they were published. Antihypertensive agents used in each study are listed in Table 1. The numbers of participants experiencing severe hypertension or superimposed pre‐eclampsia in each treatment group are denoted as “n,” with the total number of participants with chronic hypertension in each study arm denoted as “N.” Forest plots of the meta‐analysis for each maternal outcome: active vs nonactive treatment. The gray rectangles represent the risk ratio for each study and are sized in proportion to the weight assigned to the study within the analysis. The red dotted line represents to overall risk ratio for each outcome and the lateral tips of the diamond represent the 95% confidence interval for the summary measure.

Antihypertensive treatment reduces the incidence of severe hypertension in pregnancy complicated by chronic hypertension compared with no antihypertensive or placebo, with a risk ratio of 0.33 (95%CI 0.19‐0.56), based on 446 women from 5 studies. The risk of superimposed pre‐eclampsia was not significantly different between those randomized to active versus nonactive treatment; risk ratio 0.74 (95%CI 0.49‐1.11: 727 women, 7 studies) (Figure 3).

**Figure 4:**
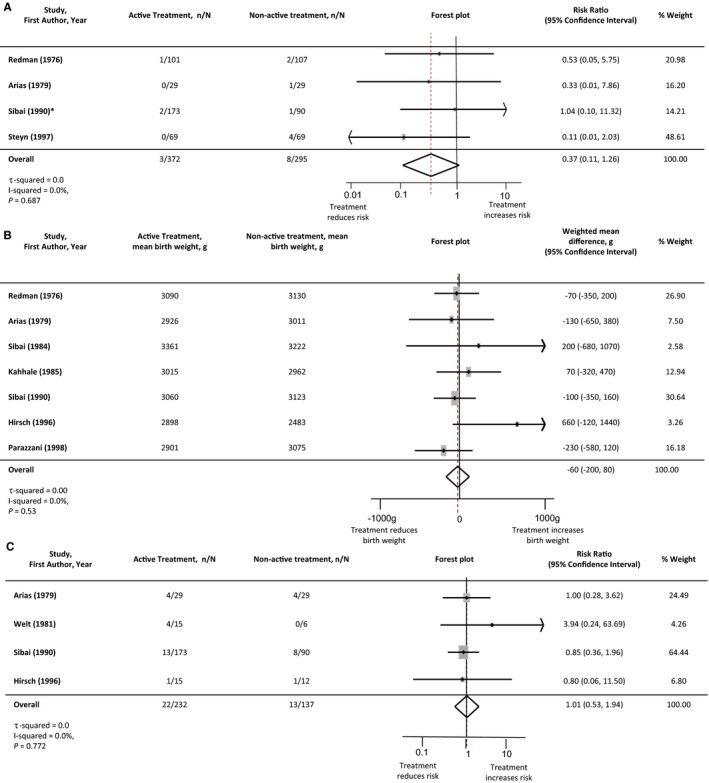
Perinatal outcomes: active vs nonactive treatment. A, Stillbirth or neonatal death. B, Birth weight. C, Small‐for‐gestational‐age infants. *Where studies had more than 1 active treatment arm, the data from the active treatment arms were pooled and compared with the nonactive treatment data. Studies are listed in order of the year they were published. Antihypertensive agents used in each study are listed in Table 1. The numbers of participants experiencing a stillbirth/neonatal death or small‐for‐gestational‐age infant in each treatment group are denoted as “n,” with the total number of participants with chronic hypertension in each study arm denoted as “N.” Forest plots of the meta‐analysis for each perinatal outcome: active vs nonactive treatment. The gray rectangles represent the risk ratio for each study and are sized in proportion to the weight assigned to the study within the analysis. The red dotted line represents to overall risk ratio for each outcome and the lateral tips of the diamond represent the 95% confidence interval for the summary measure.

**Figure 5:**
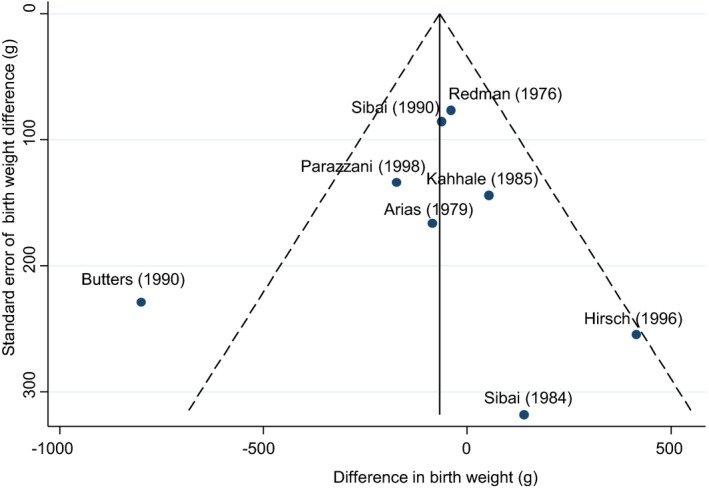
Funnel plot comparing birth‐weight difference between studies. Funnel plot demonstrates that Butters and colleagues^31^ (atenolol vs placebo) is an outlier within the meta‐analysis of birth weight when comparing active and nonactive treatment. Antihypertensive agents used in each study are listed in Table 1.

Perinatal outcomes were assessed to determine the potential fetal and neonatal risks associated with antihypertensive use when compared to nonactive treatment. The analysis of stillbirth and neonatal death demonstrated a nonsignificant reduction with the use of antihypertensive treatment: risk ratio 0.37 (95%CI 0.11‐1.26: 667 women, 4 studies). Birth weight was not significantly different when active versus nonactive treatments were compared (−60 g weighted mean difference, 95%CI −200 to 80 g: 802 women, 7 studies). There was no difference in small‐for‐gestational‐age infants with the use of antihypertensive agents (risk ratio 1.01, 95%CI 0.53‐1.94: 369 women, 4 studies) (Figure 4). A single study by Butters and colleagues comparing atenolol to placebo found a significant reduction in birth weight and increase in small‐for‐gestational‐age infants in the active treatment arm.^31^ Given the degree of heterogeneity, these results were explored further with the Egger test. This demonstrated the Butters study^31^ to be an outlier (Figure 5). When this study was included in the meta‐analysis, weighted mean difference in birth weight did not reach significance, −100 g (95%CI −240 to 40 g; I^2^ 49.6%) and similarly although the risk of small‐for‐gestational‐age birth weight increased, it was not significant (risk ratio 1.58, 95%CI l 0.88‐2.85; I^2^ 38.6%).

The additional maternal and perinatal outcomes meta‐analyzed between active and nonactive arms are listed in Table 4. There were no additional significant differences.

**Table 4:** Summary of Meta‐Analysis Findings Comparing Active With Nonactive Treatment and the Effect on Maternal and Perinatal Outcomes in Pregnancy Complicated by Chronic Hypertension

Outcome	Number of Studies Reporting Outcome	Total Participants	Risk Ratio/Weighted Mean Difference	95%CI	Degree of Heterogeneity, I^2^
Maternal					
Severe hypertension	5^30^, ^34^, ^37^, ^42^, ^45^	446	0.33	0.19 to 0.56	0.0%
Superimposed pre‐eclampsia	7^30^, ^37^, ^40^, ^41^, ^42^, ^44^, ^45^	727	0.74	0.49 to 1.11	28.1%
Cesarean section delivery	4^30^, ^37^, ^40^, ^41^	543	1.23	0.92 to 1.63	0.0%
Abruption	2^41^, ^42^	401	0.35	0.10 to 1.27	20.9%
Perinatal					
Stillbirth/neonatal death	4^30^, ^39^, ^41^, ^42^	667	0.37	0.11 to 1.26	0.0%
Birth weight, g	7^30^, ^34^, ^36^, ^38^, ^39^, ^40^, ^41^	802	−60	−200 to 80 g	0.0%
Small for gestational age	4^30^, ^34^, ^41^, ^45^	369	1.01	0.53 to 1.94	0.0%
Gestation at delivery, weeks	7^30^, ^34^, ^39^, ^40^, ^41^, ^42^, ^44^	785	0.10	−0.05 to 0.24	83.7%
Preterm birth	3^30^, ^40^, ^41^	341	1.23	0.58 to 2.54	0.0%
Apgar score <7 at 5 min	4^34^, ^36^, ^40^, ^41^	410	1.13	0.40 to 3.20	0.0%

Risk ratios provided where binary data were analyzed, and weighted mean difference given for continuous outcomes.

### Effects of Intervention: Antihypertensive Agent Versus Antihypertensive Agent

**Figure 6:**
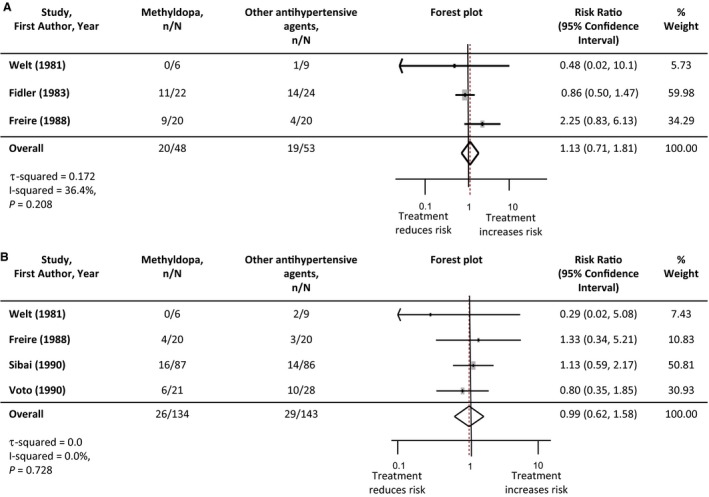
Maternal outcomes: comparison of methyldopa vs other antihypertensive agents. A, Severe hypertension. B, Superimposed pre‐eclampsia. Studies are listed in order of the year they were published. Antihypertensive agents used in each study are listed in Table 1. The number of participants experiencing severe hypertension or superimposed pre‐eclampsia in each treatment group are denoted as “n,” with the total number of participants with chronic hypertension in each study arm denoted as “N.” Forest plots of the meta‐analysis for each maternal outcome: comparison of methyldopa vs other antihypertensive agents. The gray rectangles represent the risk ratio for each study and are sized in proportion to the weight assigned to the study within the analysis. The red dotted line represents to overall risk ratio for each outcome and the lateral tips of the diamond represent the 95% confidence interval for the summary measure.

**Figure 7:**
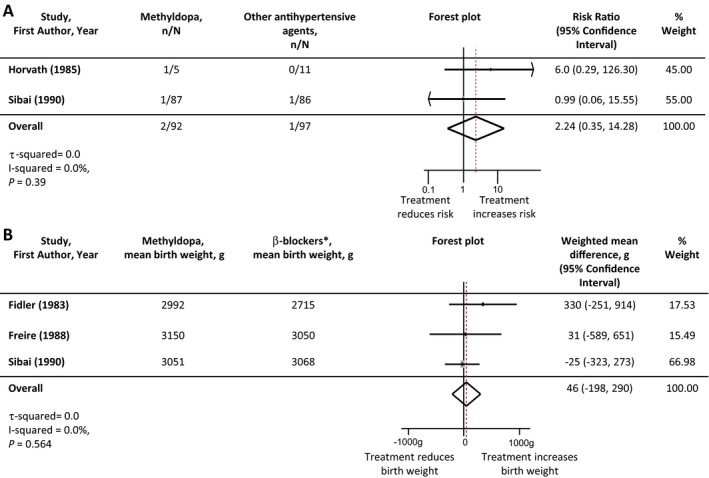
Perinatal outcomes: comparison of methyldopa vs other antihypertensive agents. A, Stillbirth and neonatal death. B, Birth weight. *Comparison made between methyldopa and beta‐blockers as these were the only agents used in head‐to‐head trials reporting birth weight. Studies are listed in order of the year they were published. Antihypertensive agents used in each study are listed in Table 1. The number of participants experiencing a stillbirth/neonatal death in each treatment group are denoted as “n,” with the total number of participants with chronic hypertension in each study arm denoted as “N.” Forest plots of the meta‐analysis for each perinatal outcome: comparison of methyldopa vs other antihypertensive agents. The gray rectangles represent the risk ratio for each study and are sized in proportion to the weight assigned to the study within the analysis. The red dotted line represents to overall risk ratio for each outcome and the lateral tips of the diamond represent the 95% confidence interval for the summary measure.

Due to the small number of studies, comparison of antihypertensive agents was restricted to methyldopa versus other classes of antihypertensive, and where possible methyldopa versus β‐blockers (Table 5). There was no difference in incidence of severe hypertension between agents when methyldopa was compared with other antihypertensive treatments. Two head‐to‐head studies (86 women) reported incidence of severe hypertension comparing methyldopa and β‐blocker antihypertensive treatment: risk ratio 0.85 (95%CI 0.57‐1.37). There was no difference in the incidence of superimposed pre‐eclampsia when methyldopa was compared with other antihypertensive agents. There were additionally no significant differences in perinatal outcomes between antihypertensive agents. Forest plots of these meta‐analyses are presented in Figures 6 and 7.

**Table 5:** Summary of Meta‐Analysis Findings Comparing Methyldopa With Other Antihypertensive Agents and the Effect on Maternal and Perinatal Outcomes in Pregnancy Complicated by Chronic Hypertension

Outcome	Number of Studies Reporting Outcome	Total Participants	Risk Ratio/Weighted Mean Difference	95%CI	Degree of Heterogeneity, I^2^
Maternal					
Severe hypertension	3^32^, ^33^, ^45^	101	1.13	0.71 to 1.81	36.4%
Superimposed pre‐eclampsia	4^33^, ^41^, ^43^, ^45^	277	0.99	0.62 to 1.58	0.0%
Perinatal					
Stillbirth/neonatal death	2^35^, ^41^	186	2.24	0.35 to 14.28	0.0%
Birth weight, g	3^32^, ^33^, ^41^	259	50	−200 to 290	0.0%

Risk ratios provided where binary data were analyzed, and weighted mean difference given for continuous outcomes.

## Discussion

This is the largest systematic review of the evidence from randomized controlled trials to guide antihypertensive treatment specifically for chronic hypertension in pregnancy. Other systematic reviews have pooled results for chronic and gestational hypertension, but given the different etiology and duration of treatment, there are concerns with this approach. The reduction in the incidence of severe hypertension in pregnant women with chronic hypertension with the use of antihypertensive treatment is clinically important given the short‐ and long‐term associated maternal morbidity and mortality.^8^, ^9^, ^10^, ^19^, ^20^, ^21^ It is not possible at this time to recommend one agent over another for optimal blood pressure control, as there have only been 3 head‐to‐head randomized controlled trials enrolling 101 women that have examined this outcome.^32^, ^33^, ^45^ No overall reduction in the risk of pre‐eclampsia was seen with the use of antihypertensive treatment.

The paucity of data to guide selection of antihypertensive treatment for chronic hypertension in pregnancy is also highlighted. Only 15 randomized controlled trials totaling 1166 pregnancies meeting study eligibility criteria were identified, and many of these were too small to address whether antihypertensive treatments reduce the risk of superimposed pre‐eclampsia or influence other measures of perinatal morbidity. The only study published in the last 18 years by Vigil and colleagues compared 3 active treatment arms and antihypertensive treatments not recommended by most international guidelines as first‐line agents (amlodipine, furosemide, and aspirin).^65^ It could not be included in the meta‐analysis given this design. This compares with many more trials and participants outside pregnancy; a recent systematic review of antihypertensive treatment (excluding pregnant participants) for the prevention of cardiovascular disease identified 123 randomized controlled trials including 613 815 participants.^69^ Of the 15 studies reported here, only 10 focused on chronic hypertension in pregnancy, and the other 5 enrolled a mixed population of chronic and gestational hypertension, from which data for the participants with chronic hypertension were extracted. Given the changes in management of hypertension both inside and outside pregnancy and that all of these trials were published between 1976 and 1998, optimal antihypertensive therapy for treating chronic hypertension in pregnancy warrants further investigation through large randomized controlled trials.

Antihypertensive use in pregnancy complicated by chronic hypertension does not increase the risk of stillbirth or neonatal death. No reduction in birth weight or increase in small‐for‐gestational‐age infants was seen, although heterogeneity was evident. This strengthens the finding that antihypertensive agents do not significantly affect perinatal morbidity; agent selection and higher than recommended dose are likely to account for the evidence from Butters and colleagues, who published data from a study of 29 participants randomized in the second trimester to atenolol or placebo.^31^ Although it is evident that the results of this study have influenced clinical practice,^8^, ^17^ this appears to be specific to this agent or to the very high doses that were used (up to 200 mg daily). Doses above 50 mg atenolol daily are not recommended and infrequently used nowadays for hypertension, as above this, the dose‐response curve is typically quite flat for blood‐pressure lowering, with the maximum licensed dose for other indications being 100 mg daily. The primary results for this analysis have been presented without the inclusion of this study for these reasons. Von Dadelszen and colleagues also analyzed with and without the data from the Butters study when examining the impact of antihypertensive treatment on the risk of small‐for‐gestational‐age newborns due to concerns over trial reporting.^16^, ^70^

Ten of the 15 studies included in the meta‐analysis evaluated agents that are no longer used for the routine management of hypertension in pregnancy in many countries (atenolol, acebutalol, oxprenolol, pindolol, bendroflumethiazide, hydrochlorothiazide, furosemide) or in the general nonpregnant population (ketanserin), accounting for about 45% of the participants studied. Although labetalol is commonly used in pregnancy, not all β‐blockers can be considered equivalent. Labetalol is a racemate with α‐ and nonselective β‐antagonist activity (in a ratio of around 1 to 3) for oral labetalol.^71^, ^72^ Oxprenolol, acebutalol, and pindolol are more selective for β_1_ receptors than β_2_ receptors but are additionally partial agonists, possessing intrinsic sympathomimetic activity (resulting in less effect on reducing heart rate). Although licensed for hypertension, β‐blockers are no longer recommended as first‐line antihypertensive treatment, but are now regarded as fourth line agents for resistant hypertension in the general (nonpregnant) population.^73^ The dose of bendroflumethiazide used (5‐10 mg daily) is higher than that currently used for hypertension (2.5 mg daily). Therefore, a substantial proportion of the evidence for treatment of hypertension in pregnancy is based on outdated drugs and outdated doses. It is difficult to draw conclusions over the effect of antihypertensive agents on other maternal and perinatal outcomes. Meta‐analysis of many maternal and fetal secondary outcomes was not possible due to a lack of reporting in the trials conducted to date. In addition, the planned adjustment for potential confounders such as body mass index was not possible due to inconsistent or absent reporting in the trial manuscripts. Further studies are needed to answer these questions and assess the potential impact of maternal characteristics such as obesity and other medical comorbidities.

The Cochrane risk‐of‐bias assessment was high or unclear for all the studies included. This is primarily due to assignment of unclear risk of bias to many areas of study conduct and restrictions in the Cochrane tool. Many studies were open‐label, assigning them high risk of bias, which reflects the difficulties in blinding medication within pregnancy when blood pressure is dynamic and multiple dosing changes are required over a short time period. Additionally, the studies are not uniform in their reported outcome measures, which reflect the large time frame and variation in geographical setting of the studies. All studies included are at least 18 years old, and given the improvements in standards of clinical care in addition to standards of study conduct, there is the potential for substantial bias to be introduced.

Previous meta‐analyses of the antihypertensive treatment of chronic hypertension in pregnancy are smaller than this study and have focused on other interventions and outcomes.^14^, ^74^ The most recent of these was published in 2000. This study aimed to assess long‐term treatment of chronic hypertension in pregnancy, and the majority of trials did not provide sufficient detail to allow categorization into mild or severe hypertension. In addition, a considerable portion of women will cross over from 1 group to the other, making analysis problematic. A Cochrane review has been conducted on the use of antihypertensive treatment for “mild to moderate” hypertension in pregnancy.^12^ The authors conclude “whether the reduction in the risk of severe hypertension is considered sufficient to warrant treatment is a decision that should be made by women in consultation with their obstetrician” and classed “mild to moderate” hypertension as a systolic blood pressure up to and including 169 mm Hg. In contrast, the Control of Hypertension in Pregnancy Study concludes that “tight control” of blood pressure should be recommended to reduce the risk of short‐ and long‐term maternal morbidity given that this does not affect fetal or neonatal outcome adversely.^18^, ^22^ Subgroup analyses of those with chronic hypertension suggest a possible trend toward small for gestational age, birth weight <10th centile (13.9% versus 19.7%; adjusted odds ratio 0.66, 95%CI 0.44‐1.00); however, it is notable that in this subgroup the primary perinatal outcome was no different (odds ratio 1.08, 95%CI 0.78‐1.51). A post hoc analysis of the Control of Hypertension in Pregnancy Study found that severe hypertension occurring in either intervention group (tight versus less‐tight control) was associated with higher rates of pregnancy loss, neonatal unit admission, and birth weight <10th centile,^22^ suggesting a perinatal benefit to reducing the risk of severe hypertension. Additionally, those with severe hypertension in the less‐tight control group were found to have an increased risk of serious maternal morbidity/mortality (odds ratio 3.74, 95%CI 1.25‐11.22).^22^ Although some still question the need to treat hypertension before it reaches severe levels, the American Heart Association and the American Stroke Association recommend systolic blood pressure should be treated above the level of 150 mm Hg to reduce the risk of stroke.^75^ This recommendation is echoed in the findings of the UK triennial enquiry into maternal death, which found severe hypertension to be a factor in a significant proportion of hypertension‐related deaths.^9^ Of note, since this recommendation, deaths from pre‐eclampsia have fallen to less than 1 per million in the UK.^76^

The potential effects of “less‐tight control” on long‐term maternal morbidity and mortality have recently been highlighted.^19^, ^20^ The Systolic Blood Pressure Intervention Trial stopped recruitment early due to the significant 25% reduction seen in a composite cardiovascular outcome (stroke, myocardial infarction, and cardiac failure) with tighter control of systolic hypertension to a target of 120 mm Hg rather than the standard treatment target of 140 mm Hg; however, this was coupled with a significant increase in serious adverse events such as hypotension, syncope, and acute kidney injury.^77^ Women of reproductive age with chronic hypertension are at substantially increased risk of cardiovascular morbidity and mortality.^78^ Reducing the incidence of severe hypertension and maintaining tighter blood pressure control in pregnancy might contribute to lowering their long‐term cardiovascular risk and warrant further investigation.

Earlier systematic reviews have focused on magnitude of initial hypertension rather than the underlying condition causing the hypertension. However, separating chronic and gestational hypertension, given the differing pathophysiological pathways and implications of treatment, allows focus on optimizing treatment for each condition and is much more relevant to clinical practice.^12^, ^13^ Advances in the understanding of the mechanisms behind the exacerbation of hypertension in pregnancy and the associated increased risk of superimposed pre‐eclampsia should be complemented with randomized controlled trials that examine how antihypertensive treatment may need to be tailored to the underlying pathophysiology. The International Society for the Study of Hypertension in Pregnancy guidelines classifying subtypes of hypertension in pregnancy have been refined over time, and head‐to‐head randomized controlled trials comparing antihypertensive agents specifically for the treatment of chronic hypertension in pregnancy using these definitions are urgently needed.^79^

There is emerging evidence that tighter control of hypertension outside pregnancy reduces risks of long‐term cardiovascular morbidity and mortality.^77^ In light of the Control of Hypertension In Pregnancy Study data suggesting fetal safety with tighter control of hypertension, future research should focus on head‐to‐head randomized controlled trials of the most commonly used antihypertensive agents in current practice; this should include smaller trials to evaluate efficacy and larger trials to assess effectiveness of agent(s) for control of chronic hypertension in pregnancy. In addition, further consideration of the impact of maternal demographic factors should be considered such as body mass index and ethnicity. Outside pregnancy, calcium‐channel blockers are recommended as first‐line antihypertensive therapy for those of African or Caribbean family origin^73^; this is due to differing pathophysiological pathways causing hypertension that vary with ethnic origin.^80^ It is possible that the efficacy of antihypertensive treatment is similarly affected by maternal ethnic background. This systematic review provides evidence to recommend that women with chronic hypertension in pregnancy should receive antihypertensive treatment to reduce the incidence of severe hypertension and its associated maternal morbidity without adversely affecting perinatal outcome.

## Conclusions

Antihypertensive treatment reduces the risk of severe hypertension in pregnant women with chronic hypertension. A considerable paucity of data exists from randomized controlled trials to guide choice of antihypertensive agent for chronic hypertension in pregnancy. Adequately powered head‐to‐head randomized controlled trials of the commonly used antihypertensive agents are required to inform prescribing.

## Author Contributions

Protocol was written by Webster and Conti‐Ramsden and reviewed by Chappell, Seed, Webb, and Nelson‐Piercy. Data were extracted and tabulated independently by Webster and Conti‐Ramsden. Data were analyzed by Seed with contributions from other authors. The first draft of the manuscript was written by Webster and Conti‐Ramsden and subsequently edited by Chappell, Webb, Seed, and Nelson‐Piercy. The guarantor of the review is Professor Lucy Chappell. All authors had full access to all of the data including statistical reports and take responsibility for the integrity of the data and accuracy of the data analysis.

## Sources of Funding

This is independent research supported by the National Institute for Health Research Professorship of Lucy Chappell (RP‐2014‐05‐019). The views expressed in this publication are those of the authors and not necessarily those of the NHS, the National Institute for Health Research, or the Department of Health. Paul Seed is partly funded by Tommy's (Registered charity no. 1060508) and Collaborations for Leadership in Applied Health Research and Care South London (National Institute for Health Research). Open access for this article was funded by King's College London.

## Disclosures

Professor Nelson‐Piercy reports personal fees from Alliance Pharmaceuticals, personal fees from UCB Pharmaceuticals, LEO Pharmaceuticals, Sanofi Aventis, and Warner Chilcott outside the submitted work. The other authors report no disclosures.

## Supporting information


**Data S1.** Study Protocol.

## Note

1 References 30, 31, 33, 34, 36, 40, 41, 42, 44, 45.
