# Supplementary material for: Impact of Antihypertensive Treatment on Maternal and Perinatal Outcomes in Pregnancy Complicated by Chronic Hypertension: A Systematic Review and Meta‐Analysis
Source: J Am Heart Assoc. 2017 May 17;6(5):e005526. doi: 10.1161/JAHA.117.005526 (PMC5524099; doi:10.1161/JAHA.117.005526)
Supplement: Supplementary file 1 — Data S1. Study Protocol. [file JAH3-6-e005526-s001.pdf]

SUPPLEMENTAL MATERIAL

Data S1.
Study Protocol

INTRODUCTION

Chronic hypertension (CHT) is estimated to affect up to 2-3% of UK pregnancies. This figure is set to increase with an ageing maternal population and the rise in obesity. Pregnancies complicated by CHT are associated with an increased risk of adverse outcomes for mother and baby. It is unclear if outcomes can be altered through choice of antihypertensive agent.

OBJECTIVES

To perform a systematic review of randomised controlled trials (RCTs) to answer the following questions:

In women with chronic hypertension in pregnancy:

- Which anti-hypertensive treatment is associated with fewest episodes of severe hypertension in pregnancy?
- Does frequency of adverse maternal outcomes (e.g. pre-eclampsia, stroke, death) and mode of delivery vary by antihypertensive agent?
- Does frequency of fetal and neonatal adverse outcomes (e.g. growth restriction, preterm delivery, neonatal unit admission) vary by antihypertensive agent?

METHODS

Population

The population is pregnant women diagnosed with chronic hypertension prior to pregnancy or diagnosed up to 20 weeks' gestation. The definitions of chronic hypertension used by each study will be tabulated. Where chronic hypertension is not described the study will be excluded. Both primary and secondary chronic hypertension will be included. Studies with intention to treat chronic hypertension, regardless of level of hypertension at study entry, will also be included. Studies in which participants had gestational hypertension (GH) or chronic hypertension will only be included if the data for the chronic hypertension population are reported separately.

Types of Intervention

- Any antihypertensive drug compared with alternative intervention (e.g. bed rest) or placebo
- One antihypertensive versus another antihypertensive drug

Eligibility criteria *(Published and unpublished RCTs in any language will be assessed for eligibility)*

- Pregnant women with chronic hypertension randomised to antihypertensive treatment arm and compared prospectively with at least one other treatment arm
- Definition of chronic hypertension reported

Exclusion criteria

- Any trial designs other than RCT.
- Studies not separating outcome data of participants with gestational or chronic hypertension

Outcomes of interest

Primary outcomes:

- Maternal
    - Severe hypertension (defined as SBP>160mmHg and/or DBP>110mmHg or as given in paper, with tabulated definitions)
- Fetal/Neonatal
    - Birthweight

Secondary outcomes:

- Maternal
    - Superimposed pre-eclampsia (with tabulated definitions)
    - Need for additional antihypertensive agent during pregnancy (enteral or parenteral)
    - Caesarean section delivery
    - Estimated blood loss at delivery
    - Eclampsia
    - HELLP syndrome (with tabulated definitions)
    - Placental abruption
    - Other severe maternal morbidity: Disseminated Intravascular Coagulation, Acute Kidney Injury, Acute Liver Injury, Stroke etc.
    - Intensive Therapy Unit/High Dependency Unit admission nights
    - Maternal death
    - Adverse events and drug side effects, including numbers withdrawn from each trial and reasons why (if available)
- Fetal/Neonatal:
    - Fetal loss: Miscarriage if <24 weeks' gestation, Stillbirth >24 weeks' gestation (or as defined)
    - Neonatal Death (death within the first 28 days of life)
    - Preterm birth (<37 weeks and subdivided into <34 weeks wherever possible)
    - Small for gestational age (SGA) babies (subdivided into birth centiles <10th centile, <3rd centile where possible)
    - APGAR score at 5 minutes
    - Arterial cord pH
    - Neonatal unit admission

- Any neonatal morbidity thought to be related to maternal antihypertensive treatment such as hypo/hypertension, hypoglycaemia, etc.

The primary outcomes have been chosen to answer the principal research question: which anti-hypertensive(s) treatment is associated with fewest episodes of severe hypertension in pregnancy? The secondary outcomes have been chosen to demonstrate any differences in maternal/fetal or neonatal outcome based on antihypertensive treatment.

Search strategy

The following databases will be searched:

- Medline (via OVID)
- Embase (via OVID)
- Cochrane Trials Register

Databases will be searched from their earliest entries until 30th April 2015. No language restriction will be used in searches. Searches will be adapted to each database and details of each planned strategy are listed in the appendix.

In addition, any currently registered relevant clinical trials will be searched for via:

- Clinicaltrials.gov
- ISRCTN.com

Other grey literature will be sought by reviewing thesis titles from WorldCat Dissertations and Theses database.

Study records and data extraction

The titles and abstracts generated from the database searches will be independently screened by two authors. If either author considers the study to meet inclusion criteria it will be included for full text assessment. Any disagreements will be resolved by involving a third independent reviewer. Any foreign language trials will be translated. Data from eligible trials will be manually extracted and entered into a standard extraction table independently by the two primary reviewers. Data from all studies will then be collectively tabulated. Where there is lack of clarity in data or study design, every effort will be made to contact the authors of the RCT for further information.

Study quality assessment

Each individual RCT will be quality assessed by one author using the Cochrane Collaboration Risk of Bias tool and then checked by a second author. Where there are any disagreements, they will be

resolved by discussion with a third author. The following areas of each study will be considered for bias:

- Sequence generation
- Allocation concealment
- Blinding of participants, personnel and outcome assessors
- Incomplete outcome data
- Selective outcome reporting
- Any other sources of bias (including funding source)

Sub-group analysis will be performed based on this quality assessment.

Data will be collected and tabulated on:

- Definition of chronic hypertension
- Clinical outcomes of interest as defined
- Criteria specified by Risk of Bias Tool
- Funding source

Data synthesis

The review will be performed in line with PRISMA guidelines. For all outcomes, the analysis will be conducted on an intention to treat basis. If a study contains only per-protocol data for a particular endpoint, it will be excluded from the main analysis. Meta-analysis will be undertaken where there is more than one study with analysable data. When there is only one study, the estimates from that study will be presented. Treatment effects will be presented as estimated differences in the mean or odds ratios with 95% confidence intervals. In the event of important significant treatment effects, the Number Needed to Treat (NNT) or Number Needed to Harm (NNH) will also be given. Conventional significance will be at the usual 5% level (2-sided).

Heterogeneity of results between studies will be tested using a Chi^2 test. Significant heterogeneity will be assessed using Tau^2 and by visual inspection of the forest plot. Where there are sufficient studies of different populations, entry criteria or treatments, meta-regression and subgroup analysis will be used to investigate the differences between study results. Meta-regression will be performed with reported variables that are suspected to influence efficacy of treatment. Subgroup analysis will be performed by:

- Ethnicity
- Age

- BMI
- Quality

Publication bias will be investigated using Egger’s test and funnel plots.

Where data collected are insufficient for quantitative synthesis, they will be tabulated for presentation and explored in the discussion. If outcomes of interest are not able to be quantitatively synthesised, recommendations for the definitive trial to obtain these data will be made in the study conclusions.

Confidence in cumulative evidence

For each outcome of interest, the quality of the body of evidence will be assessed using the Grading of Recommendations Assessment, Development and Evaluation (GRADE) system, in line with recommendations from the GRADE working group. Quality of evidence will be determined as high, moderate, low or very low through assessing: risk of bias, directness, consistency of results, precision, publication bias, magnitude of effect, and dose-response relationship. Confidence of the effect size of intervention on outcomes of interest will be based on this assessment.

APPENDIX

a. Search strategy for Medline (via OVID platform)

1. exp Hypertension/
2. exp Pregnancy/
3. exp Cardiovascular Agents/
4. 1 and 2 and 3
5. randomized controlled trial.pt.
6. controlled clinical trial.pt.
7. randomized.ab.
8. placebo.ab.
9. randomly.ab.
10. clinical trials as topic.sh.
11. trial.ti.
12. 5 or 6 or 7 or 8 or 9 or 10 or 11
13. exp animal/ not humans.sh.
14. 12 not 13
15. 4 and 14

b. Search strategy for Embase (via OVID platform)

1. exp hypertension/
2. exp pregnancy/
3. exp antihypertensive agent/
4. 1 and 2 and 3
5. random$.ab.
6. factorial$.ab.
7. crossover$.ab.
8. placebo$.ab.
9. (doubl$ adj blind$).ab.
10. (singl$ adj blind$).ab.
11. assign$.ab.
12. allocat$.ab.
13. volunteer$.ab.
14. crossover procedure/
15. double blind procedure/
16. single blind procedure/
17. exp controlled clinical trial/
18. 5 or 6 or 7 or 8 or 9 or 10 or 11 or 12 or 13 or 14 or 15 or 16 or 17
19. 4 and 18

c. Search strategy for Cochrane Library

pregnancy AND hypertension AND antihypertensive
